# Aspirin Positively Contributes to *Drosophila* Intestinal Homeostasis and Delays Aging through Targeting Imd

**DOI:** 10.14336/AD.2020.1008

**Published:** 2021-10-01

**Authors:** Yangyang Zhu, Qingshuang Cai, Xianrui Zheng, Lei Liu, Yongzhi Hua, Beibei Du, Guomin Zhao, Jiangliu Yu, Zhao Zhuo, Zhongwen Xie, Shanming Ji

**Affiliations:** ^1^Centre for Developmental Biology, School of Life Sciences, Anhui Agricultural University, Hefei, Anhui 230036, China.; ^2^State Key Laboratory of Tea Plant Biology and Utilization, School of Tea and Food Sciences and Technology, Anhui Agricultural University, Hefei, Anhui 230036, China.; ^3^Zhangzhou Affiliated Hospital of Fujian Medical University, Zhangzhou, Fujian 363000, China.; ^4^School of Life Sciences, Anhui Agricultural University, Hefei, Anhui 230036, China.; ^5^College of Animal Science and Technology, Anhui Agricultural University, Hefei, Anhui 230036, China.

**Keywords:** Aspirin, Intestinal homeostasis, Imd, K63-linked ubiquitination, *Drosophila*

## Abstract

The intestine, a high-turnover tissue, plays a critical role in regulating aging and health in both vertebrates and invertebrates. Maintaining the epithelial barrier function of the intestine by preserving innate immune homeostasis significantly delays aging and prevents mortality. In an effort to explore effective chemicals and materials that can improve intestinal integrity, we performed a nonbiased screen utilizing *Drosophila* as an animal model. We showed that long-term uptake of aspirin markedly prevented age-onset gut leakage, the over-proliferation of intestinal stem cells, and the dysbiosis of commensal microbiota in fruit flies. Mechanistically, aspirin efficiently downregulated chronic activation of intestinal immune deficiency signaling during aging. Furthermore, our *in vivo* and *in vitro* biochemical analyses indicated that aspirin is a negative modulator in control of the K63-linked ubiquitination of Imd. Our findings uncover a novel regulatory mechanism by which aspirin positively modulates intestinal homeostasis, thus delaying aging, in *Drosophila*.

Longevity is influenced by a variety of both intrinsic and extrinsic factors, which include heredity, sex, diet, health conditions, living environment, and so on [1-8]. In recent decades, numerous studies have highlighted the pivotal roles of components of the digestive tract, such as the gut, in regulating the process of aging and lifespan in both invertebrates and vertebrates [9-12]. In the aged gut, the excessive proliferation of stem cells and dysregulation of differentiated epithelial cells lead to intestinal dysplasia, which normally causes organismal mortality [10, 13-15]. Additionally, as one of the most important organs in contact with the external environment, the gut contains a certain number of symbiotic bacteria, and the bacterial load demonstrates dramatic expansion during the aging process [10, 11]. It has been suggested that the intestinal tract provides the best living environment for microorganisms; these florae and their metabolites also directly or indirectly affect many biological activities of the host, such as nutrition processing, digestion and absorption, energy balance, immune function, intestinal development and maturation [12, 16-19]. Downregulation of the association between host and commensal bacteria can cause intestinal barrier dysfunction, dysbiosis of the microbiota, diseases such as diabetes, autoimmune reactions, and even death [11, 20, 21].

Based on the great similarity of its intestine with the mammalian gut tissue and the availability of powerful genetic manipulation approaches, *Drosophila melanogaster* has emerged as a popular model organism to study the association between intestinal homeostasis and longevity. In response to the microbiota, the gut of the fruit fly mainly activates the processes of host defense and the proliferation of intestinal stem cells (ISCs), both of which involve the immune deficiency (Imd) pathway [17, 22]. Previous evidence has shown that the Imd pathway tends to become hyperactive during aging, leading to the disruption of intestinal homeostasis, which can shorten lifespan [10, 17]. Negative regulation of Imd signals can positively contribute to the turnover of ISCs, homeostasis of the microbiota, and the maintenance of epithelial barrier function and extend lifespan [11, 23].

In *Drosophila*, the Imd signaling pathway is normally activated by gram-negative bacterial infection, which results in the expression of a set of antimicrobial peptides (AMPs), such as Attacin, Cecropin and Diptericin [22]. Expression of these AMPs requires the PGRP-LC receptor in the Imd pathway and the signal-dependent cleavage and nuclear translocation of Relish, an NF-κB family transcription factor [24-26]. It has been demonstrated that ubiquitination plays a pivotal role in signal transduction in the *Drosophila* Imd pathway [22, 27]. Regulators that control the ubiquitin-related processes of key factors such as Imd [28], Dredd [29], Tak1 [30], or Relish [31] effectively determine the fate of the Imd signaling pathway and related biological activities [22, 32]. Deeper insight into these processes would reveal novel therapeutic options or medicines for the treatment of diseases and pathological conditions in mammalian systems.

To explore potential materials and molecules to help improve the epithelial barrier function of the intestine, we utilized *Drosophila* as an animal model and performed a large-scale screen of various drugs and agricultural products, such as aspirin, vitamins, strawberry slurry, and different kinds of tea extracts. We provide compelling evidence that aspirin plays a positive role in restricting age-onset gut leakage and dysbiosis of the living microbiota to delay the fruit fly aging process. Mechanistically, aspirin effectively downregulates the intestinal Imd signaling pathway by negatively controlling the K63-linked ubiquitination of Imd, thus preventing the hyperexpression of Imd-related AMPs during aging. Restriction of Imd signals in *Drosophila* intestines markedly cripples the amelioration of gut integrity and lifespan extension induced by dietary supplementation of aspirin. Thus, our study uncovers a novel Imd-dependent regulatory mechanism by which aspirin modulates *Drosophila* intestinal homeostasis and the aging process.

## MATERIALS AND METHODS

### *Drosophila* strains

All flies were reared under standard culture conditions. The *w^1118^* strain was utilized as control and host for P-element-mediated transformation. Other strains used in this study include: 1) P{*NP1-gal4*} and P{*Esg-gal4*},P{*Uasp-gfp*};P{*Tub-gal80^ts^*}, kind gifts from Dr. Yun Zhao (Shanghai Institutes for Biological Science, Chinese Academy of Sciences); 2) P{*Uasp-imd-IR(KK)*} and P{*Uasp-relish-IR(KK)*}, stocks that were obtained from Vienna *Drosophila* Resource Center; 3) *diap2^7c^*, *diap2* mutant fly that was obtained from Bloomington Stock Center; 4) P{*Uasp-artmiR-tak1*}, knockdown transgene that was obtained from Tsinghua University Fly Stock Center; 5) P{*Uasp-myc-imd*}, in which coding sequence of Myc-Imd was placed under the control of the UAS promoter.

### Cell culture and reporter assay

*Drosophila* S2 cells were cultured with insect medium (Hyclone) at 27 ?. For reporter assays, S2 cells were transfected with indicated expression plasmids along with ActinP-Renilla and AttacinP-Luciferase (Att-Luc), in which the luciferase coding sequence was placed under the *attacin* promoter. ActinP-Renilla was used as internal control. Twenty-four hours post transfection, cells were treated with indicated concentrations of aspirin for 12 h. Activities of both Firefly Luciferase and Renilla were then examined according to the manufacturer’s instructions (Promega). For data analysis, the activity of Firefly Luciferase was normalized to activity of Renilla.

### Lifespan and “Smurfs” assays

Twenty virgins (*w^1118^* or heterozygotes with indicated genotypes) were crossed to 20 indicated heterozygous males (back-crossed at least five generations into *w^1118^*). Progeny was harvested and transferred to new vials for mating. After 24 h, female flies were collected and raised on culture medium (supplemented with indicated concentrations of aspirin or not) with a density of 30 to 35 flies in each vial. Flies were transferred to new vials and counted for death every 2-day throughout adult life.

For “Smurfs” assay, the non-absorbent blue dye (FD&C blue #1) was used to analyze the intestinal barrier integrity in *Drosophila* according to standard methods as previously described [33]. In brief, indicated adult flies were transferred to a medium containing blue dye (2.5% w/v) for 2.5 h, and then the numbers of “Smurfs” were calculated.

For experimental manipulations under axenic conditions, fruit fly foods were supplemented with antibiotics including ampicillin (500 μg/mL), tetracycline (50 μg/mL), and rifamycin (200 μg/mL). Vials containing foods were autoclaved for 30 min, followed by 12 h of irradiation by a radioactive cesium source.

### Fluorescein feeding assay

To examine the potential effects of additional aspirin on the food consumption of experimental flies, we performed the Fluorescein feeding assay as previously described [34]. Briefly, flies from aspirin-treated and control groups (on the 10^th^ day) were transferred to vials with foods containing 50 μM Fluorescein (Sigma). Two hours later, 10 flies were collected (9 independent replicates for each group) and lysed with 10 mM K-phosphate buffer (pH 6.0). After centrifugation, lysates were added into 96-well plate (Corning) and analyzed in a plate reader (Tecan). The levels of Fluorescein were determined by excitation at 480 nm and emission at 521 nm, respectively. In this assay, equal volume of K-phosphate buffer was used as baseline control.

### Fly gut immunohistochemistry

Whole guts from indicated adults were dissected in 1 × phosphate-buffered saline (PBS) and fixed in fix solution (4% formaldehyde and 0.25% Tween-20 in 1 × PBS) for 30 min at room temperature, followed by washing (3 times, total 1 h) and blocking (1 h) in PBTA (0.3% Tween-20 and 1.5% BSA in 1 × PBS). Samples were incubated with indicated antibodies (diluted in PBTA) and Hoechst or DAPI to mark the nucleus, followed by observation under confocal microscopy. Primary and secondary antibodies were rabbit anti-pH3 antibodies, rabbit anti-Myc antibodies, and Alexa 555-coupled anti-rabbit antibodies, respectively.

For experiments detecting GFP signals in indicated guts, samples were directly incubated with Hoechst after treatments of fixing and blocking, followed by observation under confocal microscopy.

### Quantitative PCR assays

For bacterial *16s rRNA* gene analysis, intestines were dissected from indicated flies in sterile 1 × PBS over ice. Samples were then stored in sterile tubes at -80? overnight, followed by genomic DNA extraction utilizing the Microbiome DNA Purification Kit (Thermo). For examination of specific mRNA level, total RNA was isolated with Trizol Reagent (Invitrogen), followed by cDNA synthesis using the first-strand cDNA synthesis kit (Transgen) according to the manufacturer’s instructions. Quantitative PCR experiments were performed using SYBR Green Mix (Transgen) in triplicate on a Bio-Rad iCycler iQ5 PCR Thermal Cycler. Concentrations of specific targets were normalized to endogenous reference *rp49*. Data shown are relative levels of *16s rRNA* or specific mRNA compared with those of the control groups. Primers for quantitative PCR are listed in Table 1.

**Table 1:** Detailed information of primers used for quantitative PCR assays.

Name	Sequence
16s-F	AGAGTTTGATCCTGGCTCAG
16s-R	CTGCTGCCTYCCGTA
Diptericin-F	TTTGCAGTCCAGGGTCACCA
Diptericin-R	CACGAGCCTCCATTCAGTCCAATCTCGG
Attacin-F	CACCAGATCCTAATCGTGGCCCTGGG
Attacin-R	ACGCGAATGGGTCCTGTTGT
Cecropin A1-F	TTTCGTCGCTCTCATTCTGG
Cecropin A1-R	GACAATCCCACCCAGCTTCCCGATTGC
Imd-F	CAATCTTTGGCGGGAAGGAG
Imd-R	GGCTCCGCTGTTGTTGTTAT
Rp49-F	CACGATAGCATACAGGCCCAAGATCGG
Rp49-R	GCCATTTGTGCGACAGCTTAG

### RNA-seq analysis

*Wild-type* flies (*w^1118^*) were reared with standard foods (control) or dietary supplemented with 1 mg/L aspirin. At the age of 60^th^ day, guts were dissected, and total RNA was isolated with Trizol Reagent (Invitrogen). The purity and concentration of RNA were measured using the NanoPhotometer spectrophotometer (IMPLEN) and Qubit RNA Assay Kit in Qubit 2.0 Flurometer (Life Technologies), respectively. Samples were then sequenced and analyzed by Allewgene Technologies Co., Ltd.

### Ubiquitination assay

Transfected S2 cells or intestines were harvested and lysed in lysis buffer (50 mM Tris-HCl, pH 7.5, 150 mM NaCl, 0.5% Nonidet P-40, 10% glycerol, and 1% SDS). Samples were then boiled for 5 min and combined with binding buffer (50 mM Tris-HCl, pH 7.5, 150 mM NaCl, 0.5% Nonidet P-40, and 10% glycerol) to adjust the SDS to a final concentration of 0.1%. Lysates were subjected to sonication and immunoprecipitated utilizing anti-Flag M2 beads (Sigma, for S2 cell samples) or anti-Myc agarose beads (Abmart, for intestine samples) for 4 h, followed by washing treatment with wash buffer (50 mM Tris-HCl, pH 7.5, 500 mM NaCl, 0.5% Nonidet P-40, and 10% glycerol) for 3 times (total 1 h). Samples were loaded and subjected to western blotting assays to detect the ubiquitination levels of Imd.

**Figure 1.**
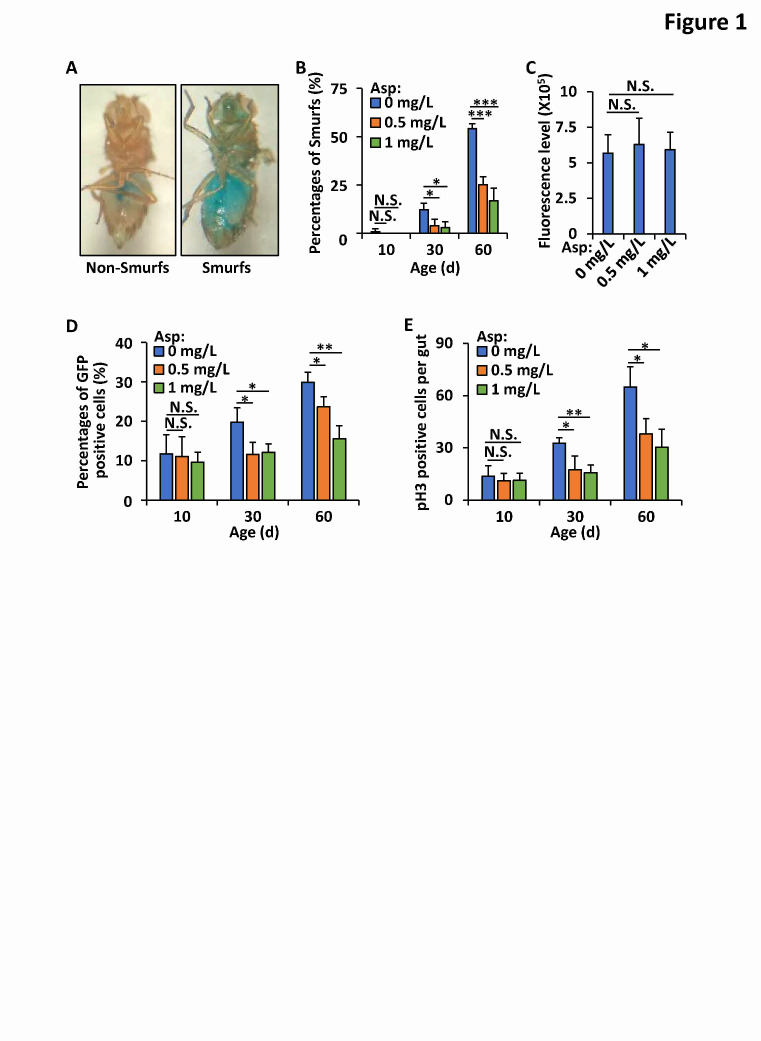
Aspirin ameliorates *Drosophila* intestinal integrity during aging. (A) *Drosophila* gut barrier function was assayed by utilizing a non-absorbent blue food dye (FD&C blue #1). Left panel, “Non-Smurfs”; right panel, “Smurfs”. (B) *Wild-type* flies (*w^1118^*) were reared with standard *Drosophila* foods (control groups), or with foods supplemented with aspirin (0.5 mg/L and 1 mg/L, respectively). At indicated ages (10-day, 30-day, and 60-day, respectively) flies from different groups were subjected to “Smurfs” assays. Proportions of flies showing gut leakage were analyzed and shown. Error bars represent s.d. (n=3). (C) *Wild-type* flies (*w^1118^*) were reared with standard *Drosophila* foods (control groups), or with foods supplemented with aspirin (0.5 mg/L and 1 mg/L, respectively). At the 10^th^ day, flies were collected and subjected to feeding assays. Levels of Fluorescein from indicated groups were monitored and shown. Error bars represent s.d. (n=9). (D) Flies genotyping P{*Esg-gal4*},P{*Uasp-gfp*};P{*Tub-gal80^ts^*} were maintained without (control groups) or with diet-supplementation of aspirin (0.5 mg/L and 1 mg/L, respectively). At indicated ages (10-day, 30-day, and 60-day, respectively) guts were dissected and subjected to immunostaining assays. Proportions of GFP positive cells per area were analyzed and shown. Error bars represent s.d. (n=3). (E) *Wild-type* Flies (*w^1118^*) were reared in the way same as in B. At indicated ages (10-day, 30-day, and 60-day, respectively) guts were dissected and subjected to immunostaining assays utilizing the antibodies against pH3. Proportions of pH3 positive cells per gut were analyzed and shown. Error bars represent s.d. (n=3). In B-E, the two-tailed Student’s t test was utilized to perform data analyses. *p<0.05, **p<0.01, ***p<0.001, N.S., not significant.

### SDD-AGE assay

The aggregation of Imd protein was examined by SDD-AGE analysis as previously described [23]. Briefly, dissected guts were lysed with lysis buffer (50 mM Tris-HCl, pH 7.5, 150 mM NaCl, 0.5% Triton X-100, 10% glycerol, and 1mM phenylmethylsulfonyl fluoride) over ice for 30 min, followed by centrifugation (13000 rpm at 4?) for 10 min. Supernatant was carefully collected and loaded with loading buffer (0.5 × TBE, 10% glycerol, 2% SDS, 0.0025% bromophenol blue) at room temperature for 15 min. Newly-prepared 1.5% agarose gel with 0.1% SDS was pre-run by electrophoresis in running buffer (1 × TBE and 0.1% SDS) for 1 h at 4?. Loaded protein samples were then subjected to electrophoresis for 1 h and transferred to PVDF membrane (Millipore) for immunoblotting analysis.

### Statistical methods

1) Two-tailed Student’s t test: data from reporter assays, quantitative PCR assays, immunostaining experiments and mean lifespan analyses were analyzed from three biological replicates; data from fluorescein feeding assay was analyzed from nine independent replicates. All the significant differences between values were determined by the two-tailed Student’s t test and statistical analyses are shown as means and standard deviations (s.d.). 2) Log rank test: the data of survival rate and protein stability assays were collected from three biological replicates and significant differences were analyzed by the Log rank test using PASW Statistics 18 software. All statistical analyses are shown as means ± s.d. For all tests, a p value of less than 0.05 was considered statistically significant. * P < 0.05, ** P < 0.01, *** P < 0.001, N.S., not significant.

**Figure 2.**
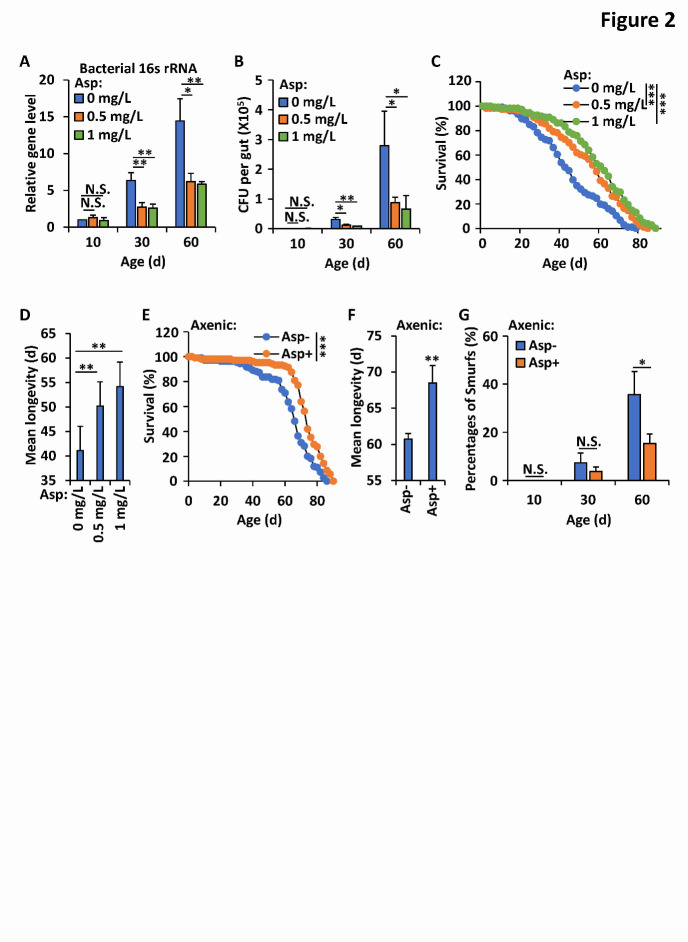
Aspirin prevents age-onset gut microbial dysbiosis and prolongs lifespan. (A) *Wild-type* flies (*w^1118^*) were reared with standard *Drosophila* foods (control), or with foods supplemented with aspirin (0.5 mg/L and 1 mg/L, respectively). Intestines were dissected from indicated adult flies at various ages (day 10, 30, and 60, respectively). Genomic DNA was extracted, followed by quantitative PCR assays of the *16s rRNA* gene to determine the commensal bacterial levels. Error bars represent s.d. (n=3). (B) *Wild-type* flies (*w^1118^*) were reared in the way same as in A. Intestinal homogenates of indicated flies were prepared and plated on nutrient-rich medium allowing growth of intestinal microbiota. CFUs per gut were further counted and shown. Error bars represent s.d. (n=3). (C-D) Flies were reared as in A, and then were collected and subjected to lifespan assays. Survival curves were analyzed and shown in c; mean longevities of samples from C were shown in D. Error bars represent s.d. (n=3). (E-G) *Wild-type* flies (*w^1118^*) were reared with standard *Drosophila* foods (Asp-) or dietary supplementation with aspirin (1 mg/L, Asp+) under axenic condition. Flies were then subjected to lifespan assay (E) or “Smurfs” assay (G) as indicated. Mean longevities of samples from E were shown in F. Error bars represent s.d. (n=3). In A, B, D, F, and G, the two-tailed Student’s t test was utilized to perform data analyses. In C and E, the log rank test was used to analyze the statistical variance of the survival rates. *p<0.05, **p<0.01, ***p<0.001, N.S., not significant.

**Figure 3.**
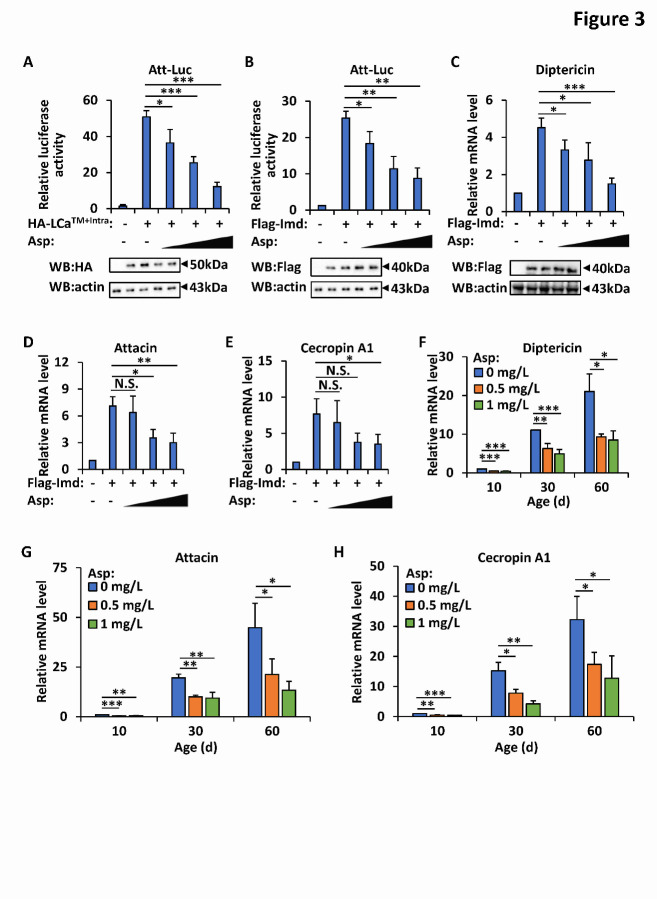
Aspirin negatively regulates Imd signaling pathway both in S2 cells and gut tissues. (A-B) S2 cells were transfected with expression plasmids of constitutively activated PGRP-LCa (A) or Imd (B) together with Att-Luc and Renilla vectors. Twenty-four hours post transfection, cells were treated with different doses of aspirin (0.1 mM, 0.5 mM, and 1 mM, respectively) for 12 h, followed by luciferase assays (upper panel) and western blotting assays with indicated antibodies (lower panels). Error bars represent s.d. (n=3). (C-E) S2 cells were transfected with Imd expression plasmids for 24 h and treated with aspirin (0.1 mM, 0.5 mM, and 1 mM, respectively) for 12 h as indicated. Cells were then lysed and subjected to qRT-PCR assays to detect the mRNA levels of *diptericin* (upper panel in C), *attacin* (D), and *cecropin A1* (E) and western blotting assays with indicated antibodies (lower panel in C). Error bars represent s.d. (n=3). (F-H) *Wild-type* flies (*w^1118^*) were reared with standard *Drosophila* foods (control), or with foods supplemented with aspirin (0.5 mg/L and 1 mg/L, respectively). Intestines were dissected from indicated groups at various ages (day 10, 30, and 60, respectively), followed by qRT-PCR assays to determine the mRNA levels of *diptericin* (F), *attacin* (G), and *cecropin A1* (H). Error bars represent s.d. (n=3). In A-H, the two-tailed Student’s t test was utilized to perform data analyses. *p<0.05, **p<0.01, ***p<0.001, N.S., not significant.

## RESULTS

### Long-term uptake of aspirin prevents age-onset intestinal barrier dysfunction

To explore functional chemicals and/or materials for the modulation of gut homeostasis, we utilized *Drosophila* as an animal model and performed noninvasive “Smurfs” experiments, in which a harmless, nonabsorbent blue dye (FD&C blue #1) was used to determine the epithelial barrier integrity of the intestine as previously described [33]. In this assay, flies in which the blue dye mainly concentrated within digestive tissues were referred to as “Non-Smurfs”; flies whose whole bodies were almost all blue were counted as “Smurfs” (Fig. 1A). First, we raised fruit flies with standard food or food supplemented with aspirin, vitamins, strawberry slurry, or tea extracts. At 10 days, 30 days, and 60 days of age, the flies were subjected to “Smurfs” assays. Interestingly, we observed that the addition of dietary aspirin to food (0.5 mg/L and 1.0 mg/L) resulted in a marked reduction in the proportion (almost 20% to 50%) of aged “Smurfs” (Fig. 1B), compared with that among the age-matched controls. These results suggest that aspirin might play a role in positively regulating the epithelial barrier function of the *Drosophila* intestine during aging. Notably, additional aspirin hardly affected the amount of food consumed by the tested flies (Fig. 1C), suggesting that the beneficial role of aspirin in preventing age-onset gut leakage was not due to dietary restriction.

### Additional aspirin ameliorates intestinal integrity during aging

We then sought to determine whether aspirin plays a role in regulating the homeostasis of the intestine. We utilized a widely used transgenic fly strain, P{*Esg-gal4*},P{*Uasp-gfp*};P{*Tub-gal80^ts^*}, in which the intestinal stem cells (ISCs) and/or enteroblasts were GFP positive [35]. Under confocal microscopy, we found that more cells were GFP positive in the gut of aged flies than in the gut of young flies (Fig. 1D), which is consistent with previous findings [33]. Moreover, we observed an almost 20% to 40% decrease in the number of GFP-positive cells in the aspirin (0.5 mg/L)-treated group compared with the controls (Fig. 1D). A further increase in aspirin (1 mg/L) resulted in a greater reduction in the number of GFP-positive cells (Fig. 1D). To further measure the proliferative integrity of the ISCs, we utilized antibodies against the phosphorylated histone H3 (pH3) protein, a marker of cell cycle progression through mitosis [36], to perform immunostaining assays. As shown in Fig. 1E, aspirin markedly delayed the over-proliferation of ISCs, as among the aged flies, the increase in the number of pH3-positive cells in the intestine was significantly prevented in the aspirin-treated group. Taken together, our results suggest that additional aspirin efficiently prevents the hyperplastic phenotype in the aged gut and limits the proliferation of intestinal progenitor cells.

### Aspirin limits age-onset expansion of the intestinal microbiota and prolongs lifespan

In *Drosophila*, age-onset dysfunction of the intestine normally correlates with microbiota imbalance and is a presage of mortality [10, 11]. Thus, we sought to determine the impacts of aspirin on commensal flora in the gut and the host lifespan. We dissected the guts of *Drosophila* at different ages with or without aspirin treatment and performed quantitative polymerase chain reaction (qPCR) assays to detect the levels of the bacterial *16s rRNA* gene, which is widely used to represent the overall extent of the commensal microbiota [37]. As shown in Fig. 2A, levels of the *16s rRNA* gene in the aspirin-supplemented groups were much lower than those in the control groups (an almost 40% to 60% reduction), suggesting that aspirin prevents age-onset expansion of the microbial community in the fruit fly intestine. Similar results were obtained when the numbers of colony-forming units (CFUs) from the gut microbiota were examined (Fig. 2B). To further detect the contribution of aspirin in controlling the *Drosophila* lifespan, we maintained experimental fruit flies in vials under standard rearing conditions and performed detailed lifespan assays as previously described [23]. As shown in Fig. 2C-D, *wild-type* adults whose food was supplemented with aspirin (0.5 mg/L and 1 mg/L, respectively) displayed a clearly prolonged lifespan compared with the lifespan of those without aspirin treatment. This was consistent with the results of previous studies [34, 38].

Then, we sought to elucidate whether aspirin prevents age-onset intestinal dysfunction and the aging process through its regulatory role in control of the gut microbiota. We performed “Smurfs” and lifespan assays utilizing *wild-type* flies under axenic conditions. As shown in Fig. 2E-G, treatment with aspirin still markedly suppressed age-onset gut barrier failure and prolonged lifespan in germ-free adult flies. Collectively, our results indicate that aspirin plays a beneficial role in controlling intestinal homeostasis and lifespan in *Drosophila*.

### Aspirin negatively regulates the Imd signaling pathway

Numerous studies have shown that Imd signaling in the *Drosophila* gut is highly related to intestinal homeostasis and longevity [11, 22, 23, 30]. The restriction of age-onset Imd signals positively contributes to the maintenance of gut barrier function and thus promotes lifespan [11, 23]. We then sought to determine whether aspirin plays a role in modulating the Imd signaling pathway. To this end, we first utilized *Drosophila* S2 cells and performed Imd signaling-based luciferase reporter assays as previously described [39]. As shown in Fig. 3A-B, the addition of aspirin dramatically decreased the luciferase activity induced by the overexpression of either constitutively activated PGRP-LCa receptor (PGRP-LCa^TM+Intra^) [40] or Imd, suggesting that aspirin might play a negative role in controlling Imd signals. Next, we performed quantitative reverse transcriptional polymerase chain reaction (qRT-PCR) assays to determine the endogenous expression profiles of the *diptericin*, *attacin*, and *cecropin A1* genes, which encode Imd-related antimicrobial peptides (AMPs). As shown in Fig. 3C-E, aspirin markedly prevented the expression of these genes in S2 cells in a dose-dependent manner.

**Figure 4.**
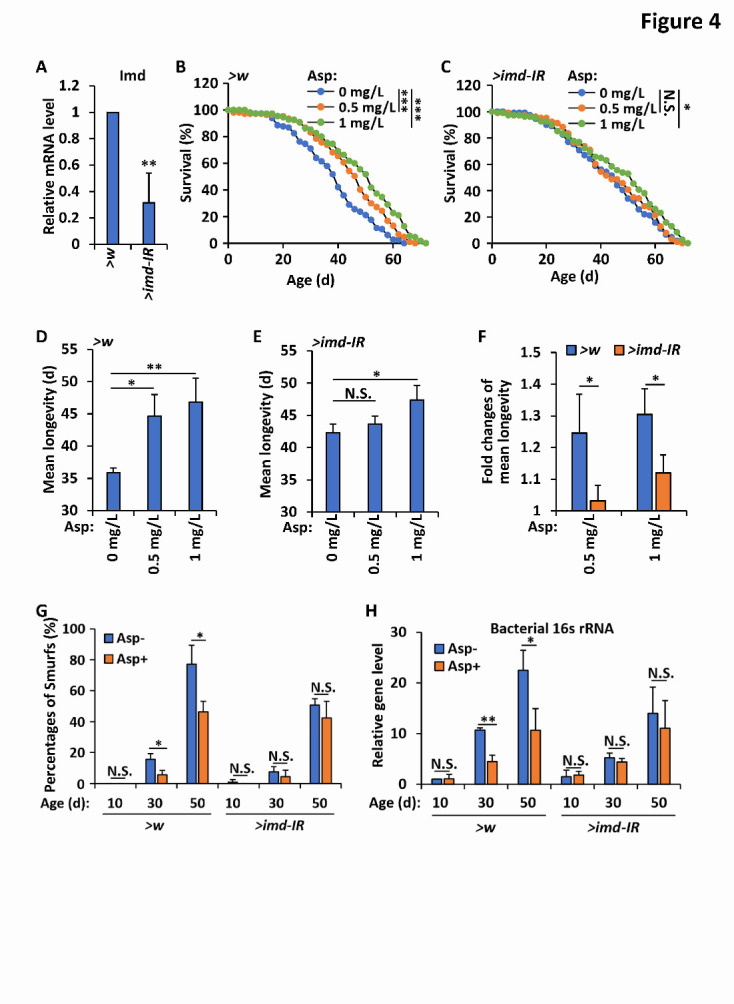
Prevention of Imd weakens advantageous effects of aspirin in modulating gut homeostasis and fly lifespan. (A) Guts of P{*NP1-gal4*} (*wild-type* controls, referred as “>*w*”) and P{*NP1-gal4*}; P{*Uasp-imd-IR(KK)*} (referred as “>*imd-IR*”) were dissected and subjected to qRT-PCR assays to detected the expressional levels of *imd*. Error bars represent s.d. (n=3). (B-F) Flies including “>*w*” and “>*imd-IR*” were reared with standard *Drosophila* foods, or with foods supplemented with aspirin (0.5 mg/L and 1 mg/L, respectively), and subjected to lifespan assays. Survival curves were analyzed and shown in B (“>*w*”) and C (“>*imd-IR*”); mean longevities of samples from B and C were shown in D (“>*w*”) and E (“>*imd-IR*”); fold changes of mean longevities of “>*w*” and “>*imd-IR*” were analyzed and shown in F. Error bars represent s.d. (n=3). (G-H) Flies including “>*w*” and “>*imd-IR*” were reared with standard *Drosophila* foods, or with foods supplemented with aspirin (0.5 mg/L and 1 mg/L, respectively). At various ages (day 10, 30, and 50, respectively) flies were subjected to “Smurfs” assays (G) or intestines were dissected from indicated adult flies, followed by genomic DNA extraction and quantitative PCR assays to determine the bacterial *16s rRNA* levels (H). Error bars represent s.d. (n=3). In A, and D-H, the two-tailed Student’s t test was utilized to perform data analyses. In B and C, the log rank test was used to analyze the statistical variance of the survival rates. *p<0.05, **p<0.01, ***p<0.001, N.S., not significant.

To further determine the role of aspirin in regulating Imd signals *in vivo*, we collected guts from flies that were fed aspirin-supplemented (0.5 mg/L and 1 mg/L) food as well as control flies at different ages and performed qRT-PCR analyses. As shown in Fig. 3F-H, among aged flies, *diptericin*, *attacin*, and *cecropin A1* transcripts in the gut were significantly downregulated in aspirin-treated flies compared with age-matched controls. Similar results were obtained when the flies were reared under axenic conditions (Fig. S1A-C). Taken together, our results suggest that aspirin likely plays a negative role in controlling the *Drosophila* Imd signaling pathway, promoting intestinal homeostasis during aging.

### Aspirin mediates intestinal homeostasis and aging in an Imd-dependent manner

To determine whether aspirin positively contributes to gut integrity and the aging process through its functional role in controlling the Imd signaling pathway, we performed genetic manipulation and obtained the progenies of P{*NP1-gal4*};P{*Uasp-imd-IR(KK)*}, in which intestinal *imd* expression was significantly prevented (Fig. 4A) [11, 41]. As shown in Fig. 4B-F, the enhancement of lifespan induced by additional aspirin in flies with the P{*NP1-gal4*};P{*Uasp-imd-IR(KK)*} genotype turned out to be much weaker than that of the *wild-type* controls, implying that aspirin positively contributes to *Drosophila* longevity, most likely in a manner dependent on Imd. Then, we performed “Smurfs” assays and found that the knockdown of *imd* preserved the age-onset reduction in intestinal leaking induced by the addition of dietary aspirin (Fig. 4G). Moreover, our quantitative PCR experiments to detect the populations of the gut microbiota showed that restricting Imd expression clearly prevented the decrease in total bacterial quantity by supplementation of the diet with aspirin (Fig. 4H).

Next, we sought to determine whether the blockade of other factors in Imd signaling would inhibit the physiological benefits of additional aspirin in foods. We used several knockdown transgenic fly strains, including P{*NP1-gal4*};P{*Uasp-artmir-tak1*} and P{*NP1-gal4*}; P{*Uasp-relish-IR(KK)*}, in which the expression of Tak1 and Relish, respectively, in the intestinal tissues was clearly prevented [23, 41]. As shown in Fig. S2A-G, downregulation of either Tak1 or Relish dramatically prevented the dietary aspirin-induced promotion of intestinal homeostasis and extension of lifespan. Consistent results were obtained when *diap2* mutant flies were utilized (Fig. S3E-H). Collectively, our genetic evidence indicates that aspirin promoted gut homeostasis and extended the *Drosophila* lifespan based on its regulatory role in controlling intestinal Imd signals.

### Aspirin is dispensable for transcriptional regulation of the Imd signaling pathway

**Figure 5.**
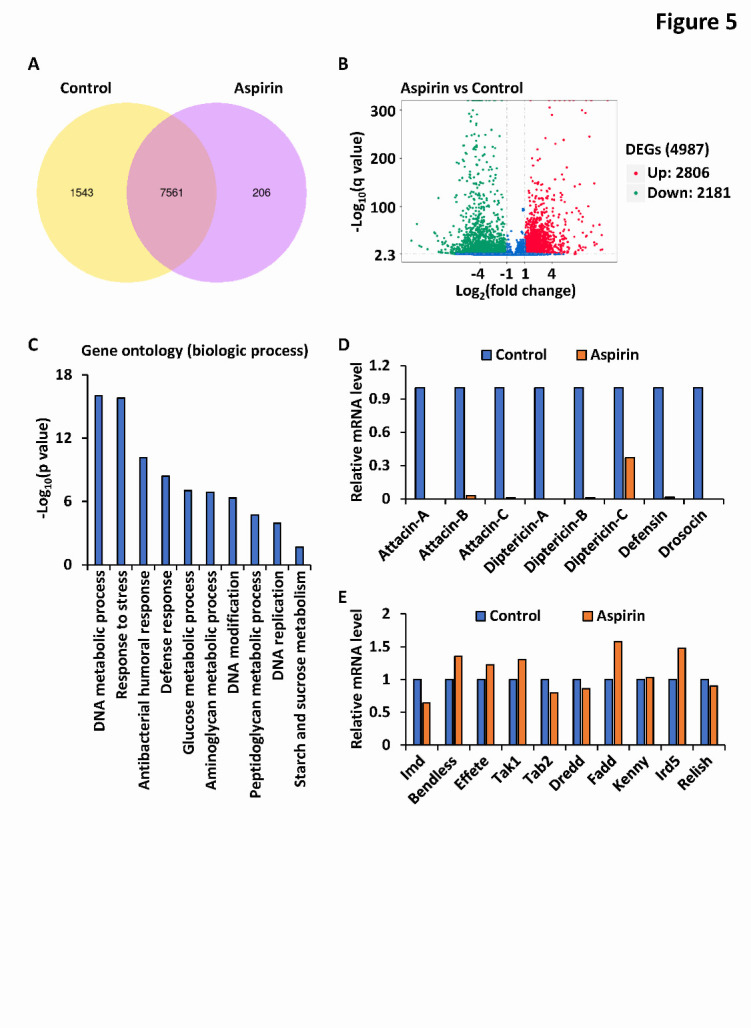
Aspirin is dispensable for transcriptional control of key factors of Imd pathway. (A-C) *Wild-type* flies (*w^1118^*) were reared with standard *Drosophila* foods (control), or with foods supplemented with aspirin (1 mg/L). On the 60^th^ day, intestines were dissected from indicated adult flies, followed by total RNA extraction and RNA-seq analysis. Venn diagram (A), volcano plots (B), and gene ontology (C) analyses of differentially expressed genes from comparisons of RNA-seq data were analyzed and shown. (D-E) Expressional levels of several AMP genes (D) and key factors in Imd pathway (E) as indicated were analyzed from RNA-seq data and shown.

Then, we sought to explore how aspirin negatively regulates the Imd signaling pathway in the *Drosophila* gut. We performed RNA-sequencing (RNA-seq) analysis utilizing the guts dissected from age-matched (60-day-old) *w^1118^* flies whose food had been supplemented without (control) or with aspirin (1 mg/L). The expression profiles of approximately 9300 genes were obtained from sequencing databases, and further bioinformatic analysis revealed that more than 80% of these genes were expressed in both groups (Fig. 5A), suggesting that aspirin does not mainly exert its effects through awakening sleeping genes. In addition, the transcript levels of almost 5000 genes were significantly altered (2806 were upregulated and 2181 were downregulated in the aspirin-treated groups compared to control groups) due to long-term aspirin uptake (Fig. 5B). Gene ontology enrichment analysis displayed that these genes were mainly involved in biological processes that include DNA metabolism, responses to stress and/or pathogenic bacteria, and so on (Fig. 5C).

Consistently, we observed that AMP genes controlled by the Imd pathway, such as *diptericin* and *attacin*, were greatly downregulated in the aspirin-treated groups (Fig. 5D). However, we failed to discover any significant alterations in the expression of core factors in Imd signaling transduction, such as *imd*, *bendless*, *effete*, *tak1*, *tab2*, *dredd*, *fadd*, *kenny*, *ird5*, and *relish* (Fig. 5E).

### Aspirin negatively modulates the K63-linked ubiquitination of Imd

**Figure 6.**
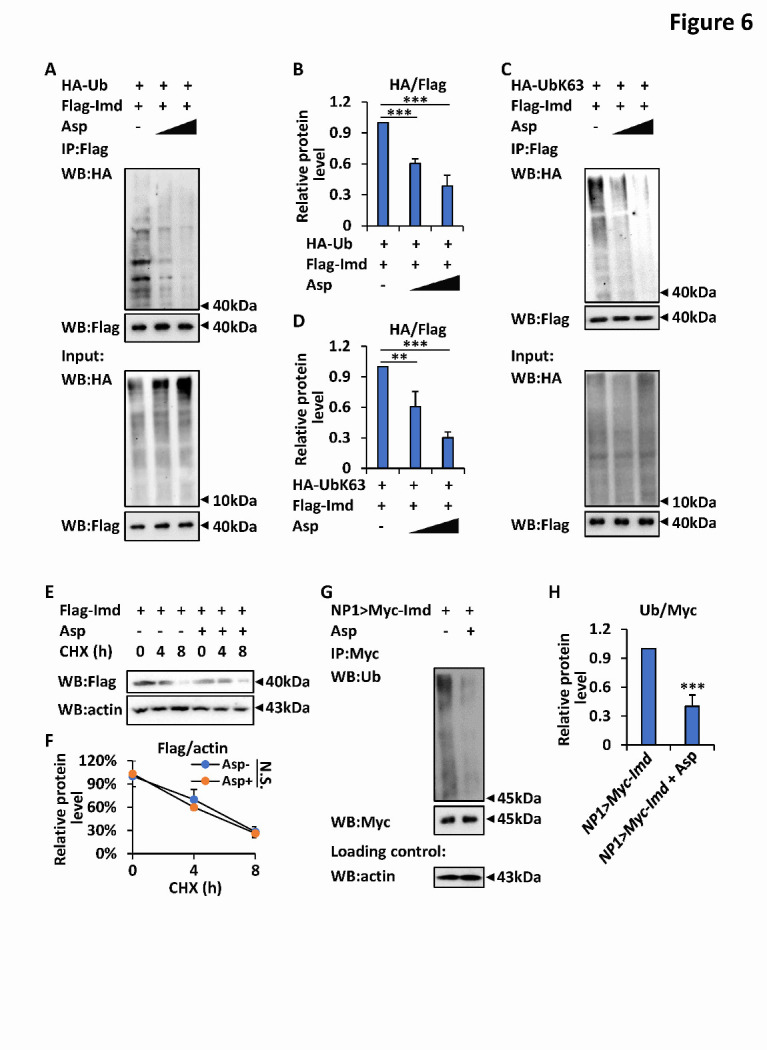
Aspirin negatively modulates K63-linked ubiquitination of Imd. (A-D) S2 cells were transfected with expression plasmids as indicated (A and C) for 36 h, and treated with different doses of aspirin (0.1 mM and 0.5 mM, respectively) or equal volume of DMSO buffer (control) for 12 h. Cells were then harvested and subjected to ubiquitination assays to monitor the levels of ubiquitination (A) or K63-linked ubiquitination (C) of Imd. Densitometry analyses to quantify intensities of ubiquitinated proteins in A and C were analyzed and shown in B and D, respectively. Error bars represent s.d. (n=3). (E-F) S2 cells were transfected with expression plasmids for 36 h and treated with or without aspirin (0.5 mM) for 12 h as indicated. Cells were then treated with CHX (final concentration at 50 ng/mL) for different times, followed by western blotting assays to assess Imd protein levels (E). Densitometry analysis to quantify Imd expression was shown in F. Error bars represent s.d. (n = 3). (G-H) Flies genotyping P{*NP1-gal4*}/P{*Uasp-myc-imd*} (referred as “*NP1>Myc- Imd”*) were reared with standard *Drosophila* foods (control), or with foods supplemented with aspirin (1 mg/L). On the 50^th^ day, intestines were dissected from indicated adult flies, followed by ubiquitination assays to determine the levels of ubiquitinated Imd (G). Densitometry analysis to quantify ubiquitination patterns of Imd was shown in H. Error bars represent s.d. (n = 3). In B, C, and H, the two-tailed Student’s t test was utilized to perform data analyses. In F, the log rank test was used to analyze the statistical variance of the protein stability. **p<0.01, ***p<0.001, N.S., not significant.

Ubiquitination plays an essential role in downstream signal transduction in the *Drosophila* Imd pathway [22, 32]. Thus, we sought to examine whether aspirin impacts the ubiquitination pattern of Imd. We transfected S2 cells with plasmids for the expression of Flag-tagged Imd and HA-tagged ubiquitin and then treated them with aspirin at different concentrations (0.1 mM and 0.5 mM, respectively). As shown by our ubiquitination assays (Fig. 6A-B), Imd ubiquitination was easily detected in the control group (treated with an equal volume of DMSO solution) and markedly decreased in the aspirin-treated groups. We then utilized another two plasmids, UbK63 and UbK48 (all lysine residues except lysine 63 and 48, respectively, had been mutated to an alanine residue) [39], and performed ubiquitination assays to detect the K63- and K48-linked ubiquitination of Imd, respectively. As shown in Fig. 6C-D, additional aspirin markedly prevented the K63-linked ubiquitination of Imd. The K48-linked ubiquitination of Imd was hardly affected by aspirin (Fig. S3A-B). Notably, aspirin was dispensable for regulating the stability of Imd in S2 cells (Fig. 6E-F).

To further confirm these results *in vivo*, we generated transgenic flies P{*NP1-gal4*}/P{*Uasp-myc-imd*}, in which the Myc-tagged Imd protein was clearly expressed in intestinal cells. We collected guts from aged P{*NP1-gal4*}/P{*Uasp-myc-imd*} flies whose diet had been supplemented with or without aspirin and performed ubiquitination assays. As shown in Fig. 6G-H, the level of Imd ubiquitination was significantly reduced in the gut samples from aspirin-treated groups compared with those from the untreated controls. When we utilized specific antibodies for K63-linked ubiquitin or polyubiquitin, we obtained similar results (Fig. S3I-J).

A previous study indicated that the amyloid formation of Imd is required for downstream signal activation and constitutes a regulatory step used by negative modulators [42]. We then sought to determine whether aspirin controls Imd activity through affecting the aggregation of Imd. Our semi-denaturing detergent agarose gel electrophoresis (SDD-AGE) experiments showed that the addition of aspirin to the fly diet hardly altered the aggregation of Imd in aged adults (Fig. S5C-D). These results were further confirmed by immunostaining assays using gut samples from aged flies (Fig. S3K). Taken together, our results suggest that aspirin negatively controls the Imd signaling pathway mainly through restricting the K63-linked ubiquitination of Imd.

## DISCUSSION

Numerous studies have highlighted the essential roles of the intestine and its living commensal microflora in aging and longevity regulation [10, 11, 43-48]. In *Drosophila*, the innate immune Imd signaling pathway has been shown to be highly correlated with intestinal homeostasis during aging [11, 22, 30]; hyperregulation of Imd signaling normally leads to dysbiosis of the gut microbiota, breakdown of the gut epithelial barrier, aging-related disorders and even death [10, 23]. Fine-tuned control of intestinal conditions by restricting Imd signals can protect the host against intestinal leakage and prolong lifespan [10, 11, 23]. In an effort to explore functional materials and/or chemicals that modulate epithelial barrier function, we performed a nonbiased screen utilizing *Drosophila* as an animal model. Intriguingly, we found that aspirin might prevent intestinal barrier dysfunction during aging since it significantly reduced the proportion of “Smurfs” flies. Further experimental approaches indicated that dietary complementation with aspirin likely inhibits age-onset expansion of the gut microbiota and intestinal dysplasia, thus delaying aging and extending lifespan in *Drosophila*. Our *in vitro* and *in vivo* biochemical analyses revealed that aspirin specifically prevents the K63-linked ubiquitination of Imd to negatively modulate Imd signal transduction and restrict the expression of downstream AMP genes. Genetic evidence has shown that downregulation of the Imd signaling pathway by the knockdown of *imd* or *tak1* or mutation of *diap2* significantly reduced the benefits of additional aspirin in promoting gut homeostasis and extending lifespan. Collectively, our results strongly suggest that aspirin promotes intestinal barrier function and delays aging through negatively controlling the intestinal Imd signaling pathway in *Drosophila*.

Previous studies have indicated that aspirin could improve the lifespan and healthspan of *Drosophila* by modulating metabolic profiles [38]. Other studies in *C. elegans* suggested that aspirin upregulated metabolic pathways to extend the lifespan in a germline-dependent manner [49]. Although our genetic evidence demonstrated that aspirin promotes gut homeostasis to prolong the longevity of *Drosophila* in a way that involves Imd signaling, RNA-seq analysis of the guts showed that additional aspirin might lead to alterations in the expression levels of several important genes that control metabolic processes, such as the glucose, peptidoglycan, starch, and sucrose metabolic pathways. In fact, we noted that restricting Imd signaling activity in the gut could only partially abrogate the extension in lifespan but nearly fully reversed the amelioration of intestinal integrity induced by additional aspirin, implying that aspirin prolongs consumer’s lifespan might through its other unknown biological functions. Nevertheless, our study demonstrates at least one novel molecular mechanism by which aspirin positively contributes to intestinal homeostasis to extend lifespan in *Drosophila*.

How does aspirin antagonize the Imd signaling pathway? Our unbiased RNA-seq analysis of the intestines from age-matched *Drosophila* treated with or without aspirin revealed no significant alterations in the transcript levels of key genes in the Imd signaling pathway, suggesting that aspirin might not be responsible for the transcriptional control of these factors. Based upon the importance of ubiquitination in signal transduction of the Imd pathway [22, 32], we performed ubiquitination assays both *in vitro* and *vivo* systems, and found that aspirin markedly prevented the K63-linked ubiquitination of Imd. However, we failed to observe any detectable variations due to the addition of aspirin when the K48-linked ubiquitination and stability of Imd were examined, although one study in Neuro A2 mouse cells suggested that aspirin can inhibit proteasome activity in a dose- and time-dependent manner, resulting in an increase in ubiquitinated proteins [50]. Because the aggregation of Imd is essential for downstream signal transduction [42] and a previous study suggested that aspirin functions to antagonize protein aggregation by promoting acetylation [51], we then detected whether aspirin affects assembly of the *Drosophila* Imd protein. Our SDD-AGE assays further indicated that additional aspirin hardly altered the levels of Imd aggregates both *in vitro* and *in vivo*. Taken together, our findings support the notion that aspirin negatively modulates the *Drosophila* Imd signaling pathway by specifically preventing the K63-linked ubiquitination of Imd.

In summary, our results indicate that dietary supplementation with aspirin dramatically ameliorated age-onset gut leakage and delayed aging in *Drosophila*. Analysis of the molecular mechanism showed that aspirin prevents the Imd signaling pathway by specifically restricting the K63-linked ubiquitination of Imd, which in turn positively contributes to maintaining homeostasis of the fly gut and extending lifespan.

## Supplementary Materials

The Supplemenantry data can be found online at: www.aginganddisease.org/EN/10.14336/AD.2020.1008.



## References

[1] Barcena C, Valdes-Mas R, Mayoral P, Garabaya C, Durand S, Rodriguez F, et al. (2019). Healthspan and lifespan extension by fecal microbiota transplantation into progeroid mice. Nat Med, 25:1234-1242. 31332389 10.1038/s41591-019-0504-5

[2] Caruso C, Accardi G, Virruso C, Candore G (2013). Sex, gender and immunosenescence: a key to understand the different lifespan between men and women? Immun Ageing, 10:20. 23680476 10.1186/1742-4933-10-20 PMC3737094

[3] Papadopoli D, Boulay K, Kazak L, Pollak M, Mallette F, Topisirovic I, et al. (2019). mTOR as a central regulator of lifespan and aging. F1000Res, 8. 10.12688/f1000research.17196.1 PMC6611156 31316753

[4] Plomion C, Aury JM, Amselem J, Leroy T, Murat F, Duplessis S, et al. (2018). Oak genome reveals facets of long lifespan. Nat Plants, 4:440-452. 29915331 10.1038/s41477-018-0172-3 PMC6086335

[5] Stolk EA, Craig BM, Mulhern B, Brown DS (2017). Health valuation: demonstrating the value of health and lifespan. The Patient, 10:515-517. 28597376 10.1007/s40271-017-0252-x

[6] Tiku V, Antebi A (2018). Nucleolar function in lifespan regulation. Trends Cell Biol, 28:662-672. 29779866 10.1016/j.tcb.2018.03.007

[7] Wildner M (2000). Gene-environment interaction and human lifespan. Lancet, 356:2103. 10.1016/S0140-6736(05)74317-8 11145526

[8] Wu Z, Isik M, Moroz N, Steinbaugh MJ, Zhang P, Blackwell TK (2019). Dietary restriction extends lifespan through metabolic regulation of innate immunity. Cell Metab, 29:1192-1205. 30905669 10.1016/j.cmet.2019.02.013 PMC6506407

[9] Akagi K, Wilson KA, Katewa SD, Ortega M, Simons J, Hilsabeck TA, et al. (2018). Dietary restriction improves intestinal cellular fitness to enhance gut barrier function and lifespan in D. melanogaster. PLoS Genet, 14:e1007777. 30383748 10.1371/journal.pgen.1007777 PMC6233930

[10] Clark RI, Salazar A, Yamada R, Fitz-Gibbon S, Morselli M, Alcaraz J, et al. (2015). Distinct Shifts in microbiota composition during Drosophila aging impair intestinal function and drive mortality. Cell Rep, 12:1656-1667. 26321641 10.1016/j.celrep.2015.08.004 PMC4565751

[11] Guo L, Karpac J, Tran SL, Jasper H (2014). PGRP-SC2 promotes gut immune homeostasis to limit commensal dysbiosis and extend lifespan. Cell, 156:109-122. 24439372 10.1016/j.cell.2013.12.018 PMC3928474

[12] Maynard C, Weinkove D (2018). The gut microbiota and ageing. Subcell Biochem, 90:351-371. 30779015 10.1007/978-981-13-2835-0_12

[13] Buchon N, Broderick NA, Chakrabarti S, Lemaitre B (2009). Invasive and indigenous microbiota impact intestinal stem cell activity through multiple pathways in Drosophila. Gene Dev, 23:2333-2344. 19797770 10.1101/gad.1827009 PMC2758745

[14] Buchon N, Broderick NA, Lemaitre B (2013 a). Gut homeostasis in a microbial world: insights from Drosophila melanogaster. Nat Rev Microbiol, 11:615-626. 23893105 10.1038/nrmicro3074

[15] Buchon N, Osman D, David FPA, Fang H, Boquete JP, Deplancke B, et al. (2013 b). Morphological and molecular characterization of adult midgut compartmentalization in Drosophila. Cell Rep, 3:1725-1738. 23643535 10.1016/j.celrep.2013.04.001

[16] Benson AK, Kelly SA, Legge R, Ma F, Low S, Kim J, et al. (2010). Individuality in gut microbiota composition is a complex polygenic trait shaped by multiple environmental and host genetic factors. P Natl Acad Sci USA, 107:18933-18938. 10.1073/pnas.1007028107 PMC2973891 20937875

[17] Bonfini A, Liu X, Buchon N (2016). From pathogens to microbiota: how Drosophila intestinal stem cells react to gut microbes. Dev Comp Immunol, 64:22-38. 26855015 10.1016/j.dci.2016.02.008

[18] Broderick NA (2016). Friend, foe or food? Recognition and the role of antimicrobial peptides in gut immunity and Drosophila-microbe interactions. Philos T R Soc B, 371:20150295. 10.1098/rstb.2015.0295 PMC4874392 27160597

[19] Wong AC, Dobson AJ, Douglas AE (2014). Gut microbiota dictates the metabolic response of Drosophila to diet. J Exp Biol, 217:1894-1901. 24577449 10.1242/jeb.101725 PMC4037322

[20] Rera M, Clark RI, Walker DW (2012). Intestinal barrier dysfunction links metabolic and inflammatory markers of aging to death in Drosophila. P Natl Acad Sci USA, 109:21528-21533. 10.1073/pnas.1215849110 PMC3535647 23236133

[21] Li X, Chiu Y, Ismail AS, Behrendt CL, Wight-Carter M, Hooper LV, et al. (2011). Mitochondrial antiviral signaling protein (MAVS) monitors commensal bacteria and induces an immune response that prevents experimental colitis. P Natl Acad Sci USA, 108:17390-17395. 10.1073/pnas.1107114108 PMC3198352 21960441

[22] Myllymaki H, Valanne S, Ramet M (2014). The Drosophila imd signaling pathway. J Immunol, 192:3455-3462. 24706930 10.4049/jimmunol.1303309

[23] Ji S, Luo Y, Cai Q, Cao Z, Zhao Y, Mei J, et al. (2019). LC domain-mediated coalescence is essential for Otu enzymatic activity to extend Drosophila lifespan. Mol Cell, 74:363-377. 30879902 10.1016/j.molcel.2019.02.004

[24] Choe KM, Lee H, Anderson KV (2005). Drosophila peptidoglycan recognition protein LC (PGRP-LC) acts as a signal-transducing innate immune receptor. P Natl Acad Sci USA, 102:1122-1126. 10.1073/pnas.0404952102 PMC545828 15657141

[25] Lemaitre B, Hoffmann JA (2007). The host defense of Drosophila melanogaster. Annu Rev Immunol, 25:697-743. 17201680 10.1146/annurev.immunol.25.022106.141615

[26] Kleino A, Silverman N (2014). The Drosophila IMD pathway in the activation of the humoral immune response. Dev Comp Immunol, 42:25-35. 23721820 10.1016/j.dci.2013.05.014 PMC3808521

[27] Thevenon D, Engel E, Avet-Rochex A, Gottar M, Bergeret E, Tricoire H, et al. (2009). The Drosophila ubiquitin-specific protease dUSP36/Scny targets IMD to prevent constitutive immune signaling. Cell Host Microbe, 6:309-320. 19837371 10.1016/j.chom.2009.09.007

[28] Paquette N, Broemer M, Aggarwal K, Chen L, Husson M, Erturk-Hasdemir D, et al. (2010). Caspase-mediated cleavage, IAP binding, and ubiquitination: linking three mechanisms crucial for Drosophila NF-kappa B signaling. Mol Cell, 37:172-182. 20122400 10.1016/j.molcel.2009.12.036 PMC2819219

[29] Meinander A, Runchel C, Tenev T, Chen L, Kim CH, Ribeiro PS, et al. (2012). Ubiquitylation of the initiator caspase DREDD is required for innate immune signalling. EMBO J, 31:2770-2783. 22549468 10.1038/emboj.2012.121 PMC3380211

[30] Fernando MDA, Kounatidis I, Ligoxygakis P (2014). Loss of Trabid, a new negative regulator of the Drosophila immune-deficiency pathway at the level of TAK1, reduces life span. PloS Genet, 10:1-13. 10.1371/journal.pgen.1004117 PMC3930493 24586180

[31] Khush RS, Cornwell WD, Uram JN, Lemaitre B (2002). A ubiquitin-proteasome pathway represses the Drosophila immune deficiency signaling cascade. Curr Biol, 12:1728-1737. 12401167 10.1016/s0960-9822(02)01214-9

[32] Zhou R, Silverman N, Hong M, Liao D, Chung Y, Chen Z, et al. The role of ubiquitination in Drosophila innate immunity. J Biol Chem, 280:34048-34055. 16081424 10.1074/jbc.M506655200

[33] Rera M, Bahadorani S, Cho J, Koehler CL, Ulgherait M, Hur JH, et al. (2011). Modulation of longevity and tissue homeostasis by the Drosophila PGC-1 homolog. Cell Metab, 14:623-634. 22055505 10.1016/j.cmet.2011.09.013 PMC3238792

[34] Danilov A, Shaposhnikov M, Shevchenko O, Zemskaya N, Zhavoronkov A, Moskalev A (2015). Influence of non-steroidal anti-inflammatory drugs on Drosophila melanogaster longevity. Oncotarget, 6:19428-19444. 26305987 10.18632/oncotarget.5118 PMC4637296

[35] Micchelli CA, Perrimon N (2006). Evidence that stem cells reside in the adult Drosophila midgut epithelium. Nature, 439:475-479. 16340959 10.1038/nature04371

[36] Amcheslavsky A, Jiang J, Ip YT (2009). Tissue damage-induced intestinal stem cell division in Drosophila. Cell Stem Cell, 4:49-61. 19128792 10.1016/j.stem.2008.10.016 PMC2659574

[37] Yarza P, Yilmaz P, Pruesse E, Glockner FO, Ludwig W, Schleifer KH, et al. (2014). Uniting the classification of cultured and uncultured bacteria and archaea using 16S rRNA gene sequences. Nat Rev Microbiol, 12:635-645. 25118885 10.1038/nrmicro3330

[38] Song C, Zhu C, Wu Q, Qi J, Gao Y, Zhang Z, et al. (2017). Metabolome analysis of effect of aspirin on Drosophila lifespan extension. Exp Gerontol, 95:54-62. 28457986 10.1016/j.exger.2017.04.010

[39] Ji S, Sun M, Zheng X, Li L, Sun L, Chen D, et al. (2014). Cell-surface localization of Pellino antagonizes Toll-mediated innate immune signalling by controlling MyD88 turnover in Drosophila. Nat Commun, 5:3458. 24632597 10.1038/ncomms4458 PMC3959197

[40] Gottar M, Gobert V, Michel T, Belvin M, Duyk G, Hoffmann JA, et al. (2002). The Drosophila immune response against Gram-negative bacteria is mediated by a peptidoglycan recognition protein. Nature, 416:640-644. 11912488 10.1038/nature734

[41] Zhai Z, Boquete JP, Lemaitre B (2018). Cell-specific Imd-NF-kappaB responses enable simultaneous antibacterial immunity and intestinal epithelial cell shedding upon bacterial infection. Immunity, 48:897-910. 29752064 10.1016/j.immuni.2018.04.010

[42] Kleino A, Ramia NF, Bozkurt G, Shen Y, Nailwal H, Huang J, et al. (2017). Peptidoglycan-sensing receptors trigger the formation of functional amyloids of the adaptor protein Imd to initiate Drosophila NF-kappaB signaling. Immunity, 47:635-647. 29045898 10.1016/j.immuni.2017.09.011 PMC5665175

[43] Salazar AM, Resnik-Docampo M, Ulgherait M, Clark RI, Shirasu-Hiza M, Jones DL, et al. (2018). Intestinal Snakeskin limits microbial dysbiosis during aging and promotes longevity. iScience, 9:229-243. 30419503 10.1016/j.isci.2018.10.022 PMC6231084

[44] He X, Yu J, Wang M, Cheng Y, Han Y, Yang S, et al. (2017). Bap180/Baf180 is required to maintain homeostasis of intestinal innate immune response in Drosophila and mice. Nat Microbiol, 2:17056. 28418397 10.1038/nmicrobiol.2017.56

[45] Thevaranjan N, Puchta A, Schulz C, Naidoo A, Szamosi JC, Verschoor CP, et al. (2017). Age-associated microbial dysbiosis promotesIntestinal permeability, systemic inflammation, and macrophage dysfunction. Cell Host Microbe, 21:455-466. 28407483 10.1016/j.chom.2017.03.002 PMC5392495

[46] Douglas AE (2019). Simple animal models for microbiome research. Nat Rev Microbiol, 17:764-775. 31417197 10.1038/s41579-019-0242-1

[47] Iatsenko I, Boquite JP, Lemaitre B (2018). Microbiota-derived lactate activates production of reactive oxygen species by the intestinal NADPH oxidase Nox and shortens Drosophila lifespan. Immunity, 49:929-942. 30446385 10.1016/j.immuni.2018.09.017

[48] Rodriguez-Fernandez IA, Qi Y, Jasper H (2019). Loss of a proteostatic checkpoint in intestinal stem cells contributes to age-related epithelial dysfunction. Nat Commun, 10:1050. 30837466 10.1038/s41467-019-08982-9 PMC6401111

[49] Huang X, Mu X, Wan Q, He X, Wu G, Luo H (2017). Aspirin increases metabolism through germline signalling to extend the lifespan of Caenorhabditis elegans. PloS One, 12:e0184027. 28910305 10.1371/journal.pone.0184027 PMC5598954

[50] Dikshit P, Chatterjee M, Goswami A, Mishra A, Jana NR (2006). Aspirin induces apoptosis through the inhibition of proteasome function. J Biol Chem, 281:29228-29235. 16880202 10.1074/jbc.M602629200

[51] Ayyadevara S, Balasubramaniam M, Kakraba S, Alla R, Mehta JL, Reis RJS (2017). Aspirin-mediated acetylation protects against multiple neurodegenerative pathologies by impeding protein aggregation. Antioxid Redox Sign, 27:1383-1396. 10.1089/ars.2016.6978 PMC5661865 28537433

