# Supplementary material for: Aspirin Positively Contributes to Drosophila Intestinal Homeostasis and Delays Aging through Targeting Imd
Source: Aging Dis. 2021 Oct 1;12(7):1821–34. doi: 10.14336/AD.2020.1008 (PMC8460307; doi:10.14336/AD.2020.1008)
Supplement: Supplementary file 1 [file AD-12-7-1821-s.pdf]

SUPPLEMENTARY DATA

Aspirin Positively Contributes to *Drosophila* Intestinal Homeostasis and Delays Aging through Targeting Imd

Yangyang Zhu[1,#], Qingshuang Cai[2,#], Xianrui Zheng[3], Lei Liu[1], Yongzhi Hua[1], Beibei Du[1], Guomin Zhao[1], Jiangliu Yu[4], Zhao Zhuo[5], Zhongwen Xie[2], Shanming Ji[1*]

© 2020. Zhu Y et al. Published online at http://www.aginganddisease.org/EN/10.14336/AD.2020.1007

SUPPLEMENTARY DATA

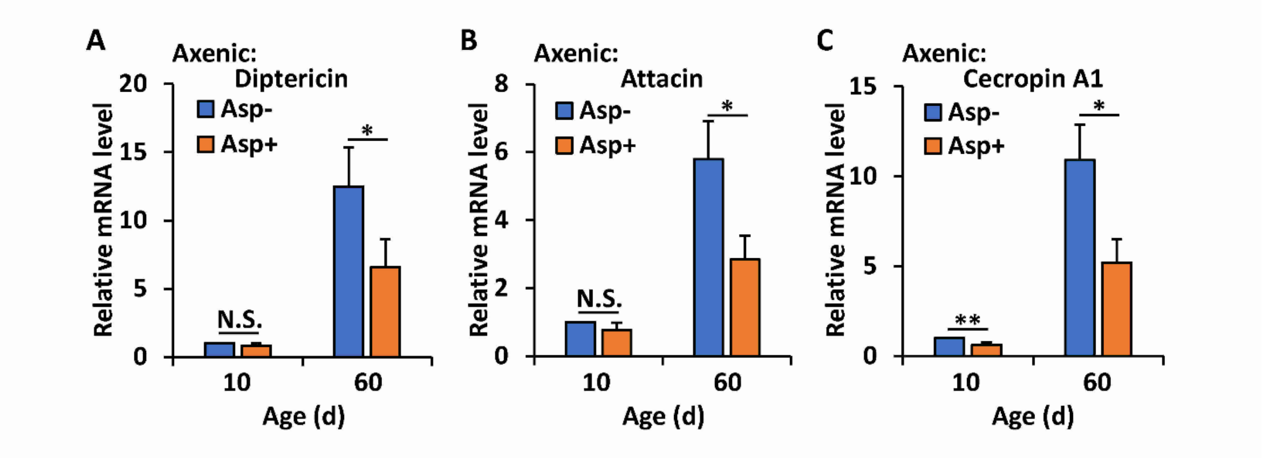

Supplementary Figure 1. Dietary supplementation of aspirin reduces the mRNA levels of intestinal AMP genes under axenic condition. (A-C) *Wild-type* (w^{1118}) flies were reared with standard *Drosophila* foods (control) or dietary supplementation with aspirin (1 mg/L) under axenic condition. At indicated ages (10-day and 60-day, respectively), guts were dissected and subjected to qRT-PCR assays to monitor the mRNA levels of *diptericin* (A), *attacin* (B), and *cecropin A1* (C). Error bars represent s.d. (n=3). The two-tailed Student's t test was utilized to perform data analyses. *p<0.05, **p<0.01, N.S., not significant.

© 2020. Zhu Y et al. Published online at http://www.aginganddisease.org/EN/10.14336/AD.2020.1007

SUPPLEMENTARY DATA

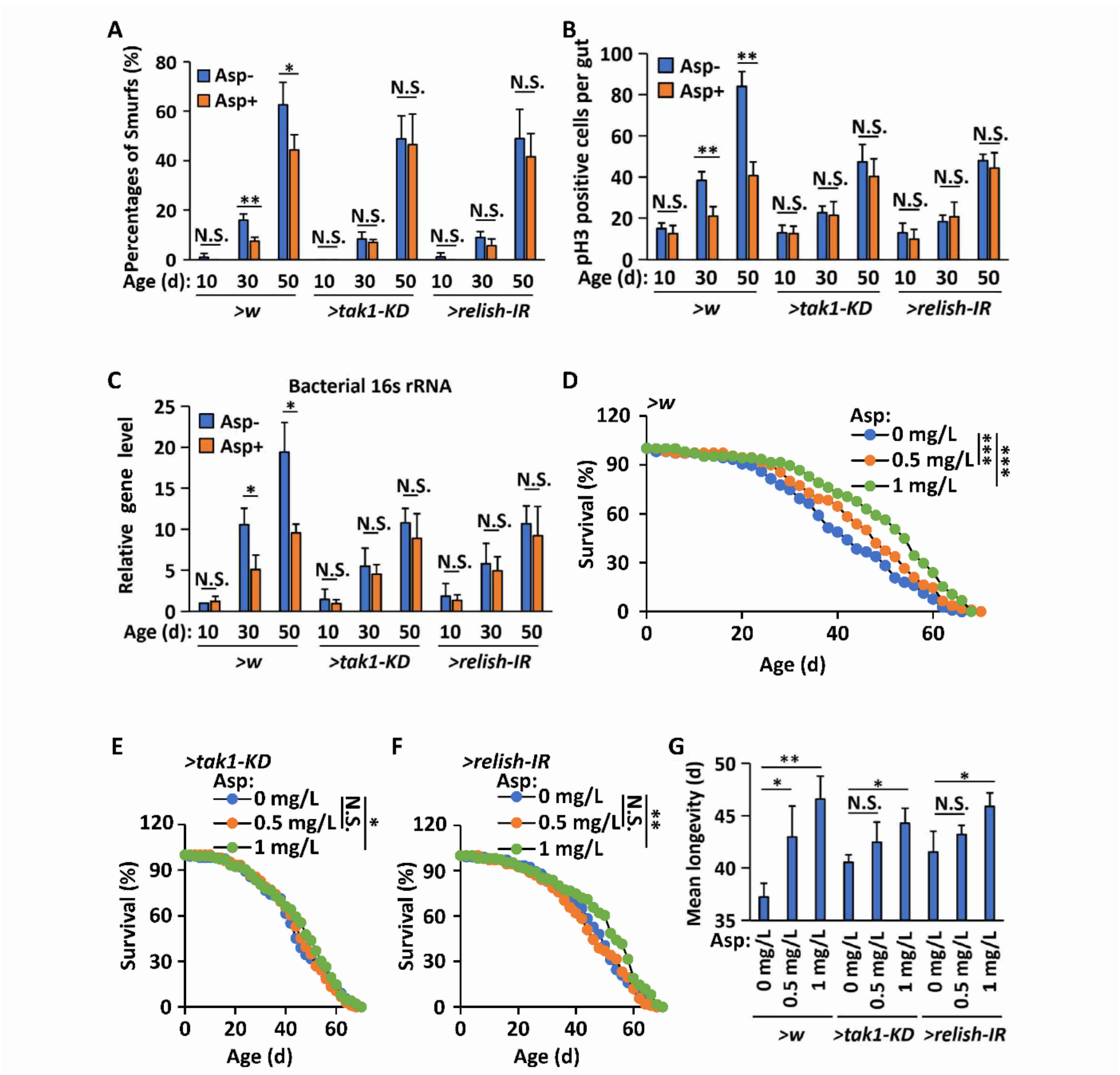

Supplementary Figure 2. Knocking down of *tak1* or *relish* prevents amelioration of gut integrity by additional aspirin. (A) Indicated flies were reared with standard *Drosophila* foods (control) or dietary supplementation with aspirin (1 mg/L). At indicated ages (10-day, 30-day, and 50-day, respectively), flies were subjected to “Smurfs” assays. Proportions of “Smurfs” flies were analyzed and shown. Error bars represent s.d. (n=3). **(B)** Flies were reared as in A. At indicated ages (10-day, 30-day, and 50-day, respectively), guts were dissected and subjected to immunostaining assays. Proportions of pH3 positive cells per gut were analyzed and shown. Error bars represent s.d. (n=3). **(C)** Flies were reared as in A. At indicated ages (10-day, 30-day, and 50-day, respectively), guts were dissected, followed by genomic DNA extraction and quantitative PCR assays to determine the levels of bacterial 16s rRNA. Error bars represent s.d. (n=3). **(D-G)** Flies were reared as in A, and then were collected and subjected to lifespan assays. Survival curves were analyzed and shown in D-F; mean longevities of samples from D-F were shown in G. Error bars represent s.d. (n=3). In A-C and G, the two-tailed Student’s t test was utilized to perform data analyses. In D-F, the log rank test was used to analyze the statistical variance of the survival rates. *p<0.05, **p<0.01, ***p<0.001, N.S., not significant.

© 2020. Zhu Y et al. Published online at http://www.aginganddisease.org/EN/10.14336/AD.2020.1007

SUPPLEMENTARY DATA

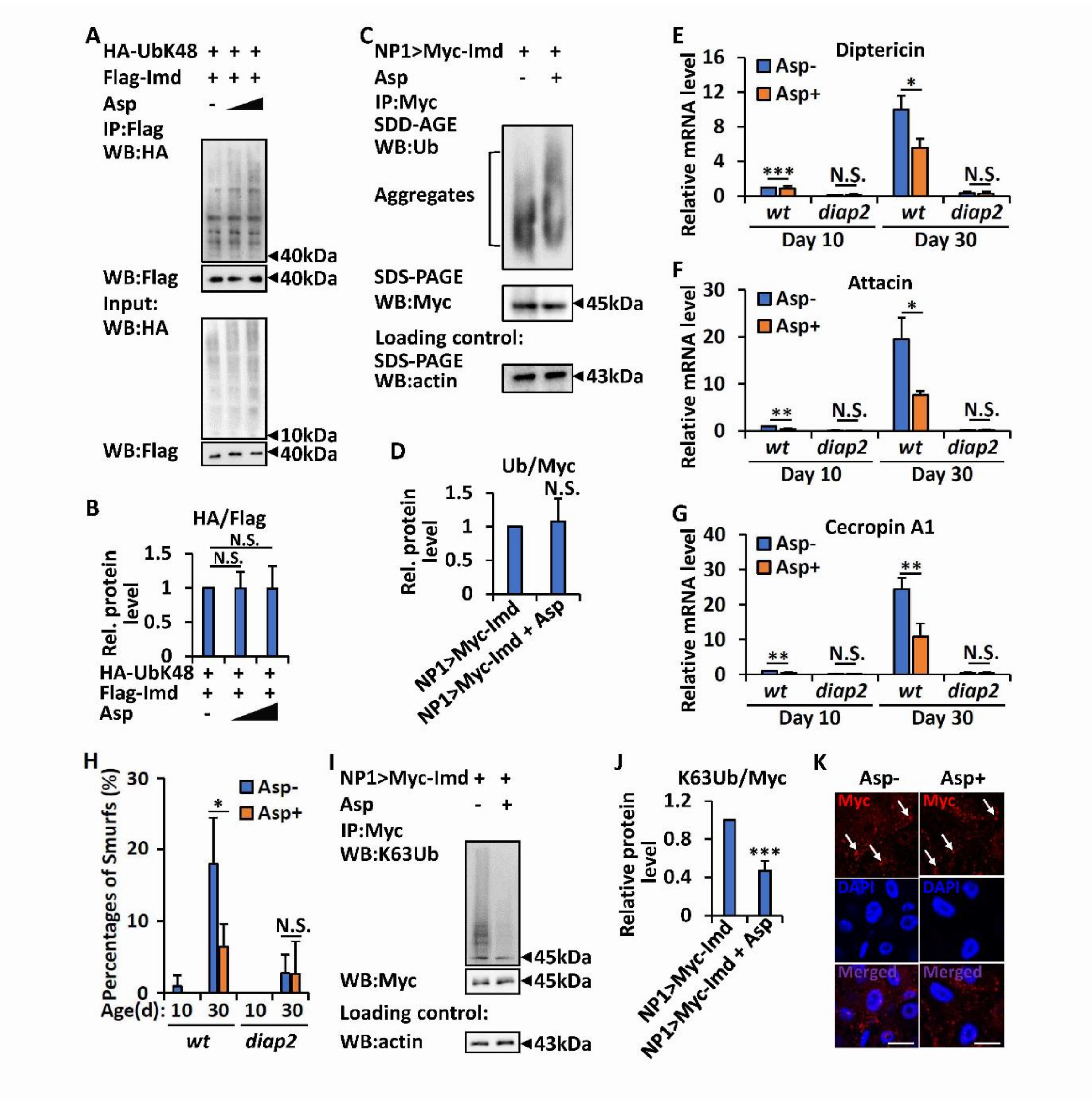

Supplementary Figure 3. Aspirin is dispensable for modulating the K48-linked ubiquitination or aggregation of Imd. (A-B) S2 cells were transfected with expression plasmids as indicated for 36 h, and treated with different doses of aspirin (0.1 mM and 0.5 mM) or buffer (control) for 12 h. Cells were then harvested and subjected to ubiquitination assays (A). Densitometry analyses to quantify intensities of K48-linked ubiquitination of Imd proteins in (A) were analyzed and shown in (B). Error bars represent s.d. (n=3). **(C-D)** Flies genotyping P{*NP1-gal4*}/P{*Uasp-myc-imd*} (referred as *NP1>Myc-Imd*) were reared with standard *Drosophila* foods (control), or with foods supplemented with aspirin (1 mg/L). On the 60th day, intestines were dissected and lysed, followed by SDD-AGE assays to determine the aggregation patterns of Imd (C). Densitometry analysis to quantify Imd aggregates was shown in (D). Error bars represent s.d. (n = 3). **(E-H)** *Wild-type* (w^{1118}) and *diap2* mutant flies were reared with standard *Drosophila* foods (Asp-), or with foods supplemented with aspirin (1 mg/L, Asp+). At indicated ages, guts were dissected and subjected to qRT-PCR assays to determine the mRNA levels of *diptericin* (E), *attacin* (F), and *cecropin A1* (G); or flies were subjected to "Smurfs" assays (H). Proportions of flies showing gut leakage were analyzed and shown in H. Error bars represent s.d. (n=3). **(I-J)** *NP1>Myc-Imd* flies were reared with standard *Drosophila* foods (control), or with foods supplemented with aspirin (1 mg/L). On the 30th day, intestines were dissected and subjected to ubiquitination assays using antibodies against K63-linked ubiquitin or poly-ubiquitin (I). Densitometry analyses to quantify intensities of K63-linked ubiquitination of Imd proteins in (I) were analyzed and shown in (J). Error bars represent s.d. (n=3). **(K)** *NP1>Myc-Imd* flies were reared same as in I. On the 30th day, intestines were dissected and subjected to immunostaining assays utilizing antibodies against Myc. DAPI was used to mark the nucleus. Scale bars, 10 µm. In B, D, E-H, and J, the two-tailed Student's t test was utilized to perform data analyses. *p<0.05, **p<0.01, ***p<0.001, N.S., not significant.

© 2020. Zhu Y et al. Published online at http://www.aginganddisease.org/EN/10.14336/AD.2020.1007
